# Daylight Bactericidal Titania Textiles: A Contribution to Nosocomial Infections Control

**DOI:** 10.3390/molecules24101891

**Published:** 2019-05-16

**Authors:** Joana C. Matos, Cláudia Oliveira, M. Clara Gonçalves

**Affiliations:** 1 Departamento de Engenharia Química, Instituto Superior Técnico, Universidade de Lisboa, 1049-001 Lisboa, Portugal; joanacmatos@gmail.com; 2 CQE, Centro de Química Estrutural, Instituto Superior Técnico, Universidade de Lisboa, 1049-001 Lisboa, Portugal; 3 Departamento de Biologia, CESAM, Universidade de Aveiro, Campus Universitário de Santiago, 3810-193 Aveiro, Portugal; csoliveira@ua.pt

**Keywords:** nanoparticles, amorphous titania, chitosan, bactericidal, ASTM E2149, AATCC 100, *Staphylococcus aureus*

## Abstract

Daylight bactericidal cotton (100% cotton) textiles are presented and proposed for future hospital use. Amorphous titania (a-TiO_2_) and amorphous titania/chitosan complexes (a-TiO_2_//CS) were the selected bactericidal agents. Nanoparticles (NPs) and films were the two paths designed. Cotton textiles were impregnated with a-TiO_2_-based NPs or coated with a-TiO_2_ films. Industrial impregnation/coating will be implemented during the textile finishing treatments. A novel (room temperature and base-catalyzed), green (hydrothermal water as a catalyst), time-saving, and easy scale-up sol–gel process was established to produce the a-TiO_2_-based NPs. Amorphous-TiO_2_ films were produced by a dip-in (acid catalyzed) sol–gel solution. The daylight bactericidal performance (without the need of an external ultraviolet light source) of a-TiO_2_ NPs, films, and impregnated/coated textiles was proven according to AATCC 100 and ASTM E2149, using *Staphylococcus aureus* (ATCC^®^6538^TM^) as the bacterial indicator strain. A bacterial reduction of 99.97% was achieved for the a-TiO_2_ films and of 99.97% for the a-TiO_2_//CS NPs. Regarding the impregnated textiles, a bacterial reduction of 91.66% was achieved with a-TiO_2_//CS NPs, and 99.97% for cotton textiles coated with an a-TiO_2_ film.

## 1. Introduction

Nosocomial infections, otherwise known as healthcare-associated infections (HCAIs), occur worldwide both in developed and developing countries, affecting approximately 8.7% of all hospitalized patients (7% in developed and 10% in developing countries), which causes prolonged hospital stay, disability, and unacceptable costs to healthcare economies [1]. According to the European Centre for Disease Prevention and Control (ECDC), three million cases of nosocomial infections occur annually, 50,000 of which are fatal [2]; thus, its control is a worldwide health priority [1,2,3]. Still, the growth of bacterial resistance is rendering antimicrobial agents less effective, while nosocomial pathogens (bacteria, viruses, and fungi) contaminate the surfaces and equipment handled by hospital staff, with hospital textiles being an important source of cross infections. One way to stop this dissemination is to give to textiles bactericidal properties. Thus, in order to impart them with bactericidal activity, different approaches have been purposed [4], as follows: (i) the inclusion of antimicrobial compounds in the polymeric fibers, (ii) grafting of certain moieties onto the polymer surface, or (iii) physical modification of the textile during finishing processes—the route adopted in the present work.

**Figure 1:**
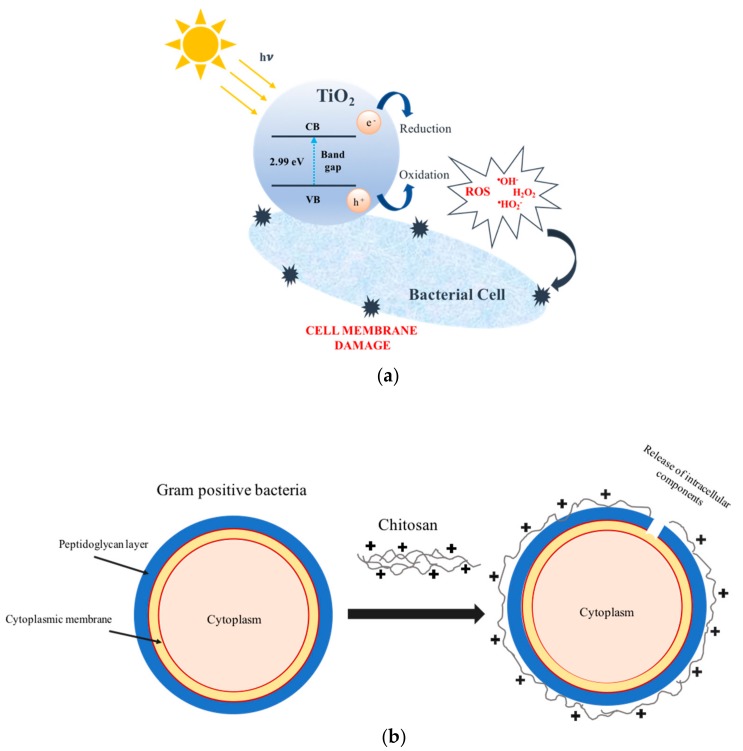
Damage to bacterial cell membranes induced by the following: (**a**) reactive oxygen species (ROS) generated when crystalline titania (TiO_2_) is imposed to daylight and (**b**) chitosan polymer. Note: VB-valence band, CB-conduction band.

Oxide semiconductors, acting as photocatalysts, play a catalytic role in a wide range of cell enzymes, either generating or catalyzing reactive oxygen species (ROS), which can induce oxidative stress, damaging cellular proteins, lipids, and DNA [5]. Crystalline TiO_2_ nanoparticles (NPs) (approved by the American Food and Drug Administration for use in human food, drugs, cosmetics, and food contact materials, [6]) excel because of their photo-reactivity, photostability, reusability, inexpensively, non-toxicity, and effective bactericidal performance, which are promising for eliminating microorganisms in self-cleaning and self-sterilizing materials [7,8,9]. When crystalline TiO_2_ is exposed to ultraviolet light (λ < 400 nm), photo-excited holes (h_vb_^+^) and electron (e_cb_^−^) charge carriers are generated. Within nanoseconds, ≈90% of e_cb_^−^−h_vb_^+^ pairs recombine without any chemical effect (simply by dissipating the absorbed *hv* energy as heat), while ≈8% migrate to the defects in the crystalline network (a second decay mechanism [5,6]). Only the remaining 1%–2% fraction of photo-generated charges can promote chemical reactions with surface adsorbed molecules, either generating or catalyzing ROS [5,6] (Figure 1a), which start by damaging the bacterial cells’ surfaces weak points, and gradually increase cell permeability. This permeability allows for the free efflux of intracellular contents, causing cell death [10,11,12,13,14,15,16,17,18,19].

Chitosan (CS) also presents bactericidal properties. This is a versatile hydrophilic polysaccharide of animal origin found abundantly in nature and characterized by a fibrous structure. The bactericidal mechanism of CS is based on cationically-charged amino groups (−NH^3+^) of glucosamine that may combine with anionic components on the Gram-positive bacteria cell surface, and may inhibit enzymes, resulting in extensive cell surface attraction, a leakage of intracellular substances, and the suppression of bacteria growth ending by damaging vital bacterial cell activities [20] (Figure 1b). Chitosan’s biocompatibility, non-toxicity, non-carcinogenicity, and antimicrobial activity allows for its use in commercial applications, such as food preservation, dentistry, and ophthalmology, and in the manufacture of wound-dressings and antimicrobial-finished textiles, namely, in cotton, polyester, and wood fibers, where patented products have reached the market (Crabyon^®^ OMIKENSHI, Japan) [21,22,23,24,25,26,27]. CS amine groups present a high affinity to titania, being commonly used as a stabilizing agent for titania NPs in (water) dispersions [28]. The ultraviolet activated chitosan-(crystalline) titania bactericidal synergy has been reported in applications such as catalyst [29], protective films [30], filtration membranes [31], and biomaterials, to use as implants or in tissue engineering products [32].

This work presents a-TiO_2_-based NPs (pristine a-TiO_2_, a-TiO_2_-NH_2_, and a-TiO_2_//CS) synthesized by a novel, room-temperature, base-catalyzed, green (ammonia-free) sol–gel protocol. TiO_2_ films were also used, which were synthesized by a classical acid catalyzed sol–gel process [33]. NP and film morphology, a lack of crystallinity, and structural characterization were performed through transmission electron microscopy (TEM), X-ray analysis (XRD), and Fourier transform infrared spectroscopy (FTIR). The daylight bactericidal properties (without the need for an external ultra-violet light source) of the obtained NPs/films were evaluated according the ASTM E2149 test method. The second part of this work illustrates the application of a-TiO_2_ products to the textile industry, where a cost-effective, easy scale-up process was developed, and a daylight bactericidal commodity was achieved. The daylight bactericidal performance (without the need of external ultra-violet light source) of impregnated or coated textiles with titania NPs/films, was evaluated according to the AATCC 100 test method, using *Staphylococcus aureus* (*S. aureus*, ATCC^®^6538^TM^) as reference test strain.

## 2. Materials and Methods

### 2.1. Materials

All chemicals—aqueous sodium silicate solution (SSS; Na_2_O.SiO_2_, 27% wt. % SiO_2_), titanium IV isopropoxide (TiPOT, 97%), 3-aminopropyltriethoxysilane (APTES; 99%), tetraethyl orthosilicate (TEOS; ≥99%), chitosan (50,000 to 190,000 Da, 75%–85% deacetylate), and nitric acid (HNO_3_ ≥ 65%)—were purchased from Sigma-Aldrich (Darmstadt, Germany), and were used without further purification. Absolute ethanol (EtOH; 99.5%), from Merck (Darmstadt, Germany); bi-distilled water (conductivity 0–2 µS/cm^3^, pH 5.8–6.5); and hydrothermal water (SPA, Cabeço de Vide, Portugal) were also used. Acetic acid glacial (CH_3_COOH; 99+%) was purchased from Alfa Aesar (Ward Hill, USA), and hydrochloric acid was purchased (HCl, 35%–38%) from Sigma-Aldrich (Darmstadt, Germany),

Cotton (100% cotton) fabric, 120 g/m^2^ basis weight, was supplied by CITEVE, Portugal. Microbiology culture media, Luria broth (LB), and Luria agar (LA) were purchased from VWR (USA).

### 2.2. Experimental Methodology

**Figure 2:**
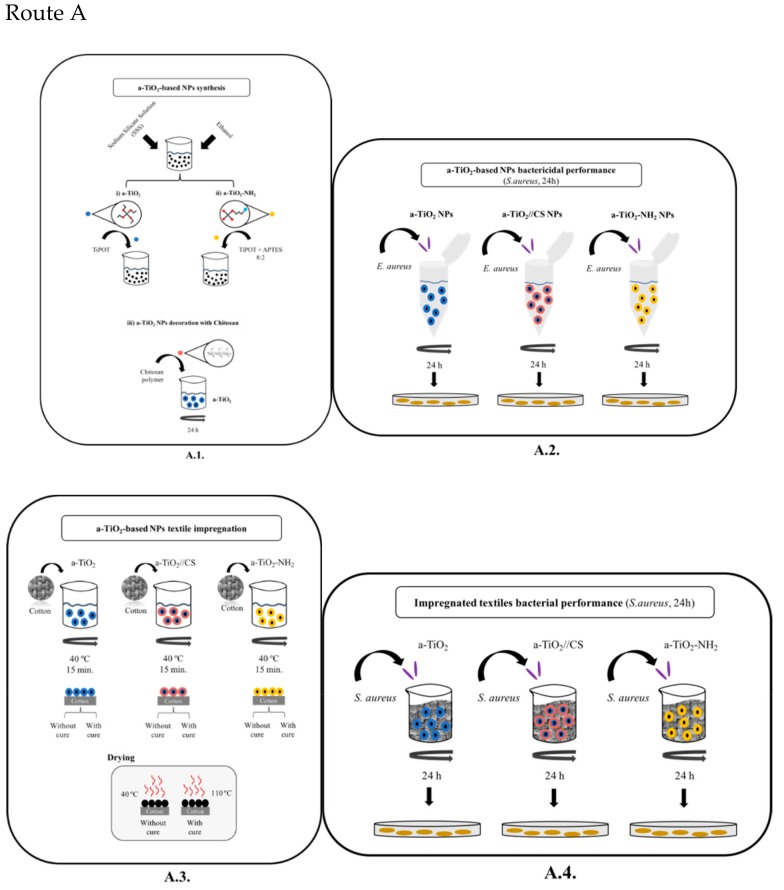

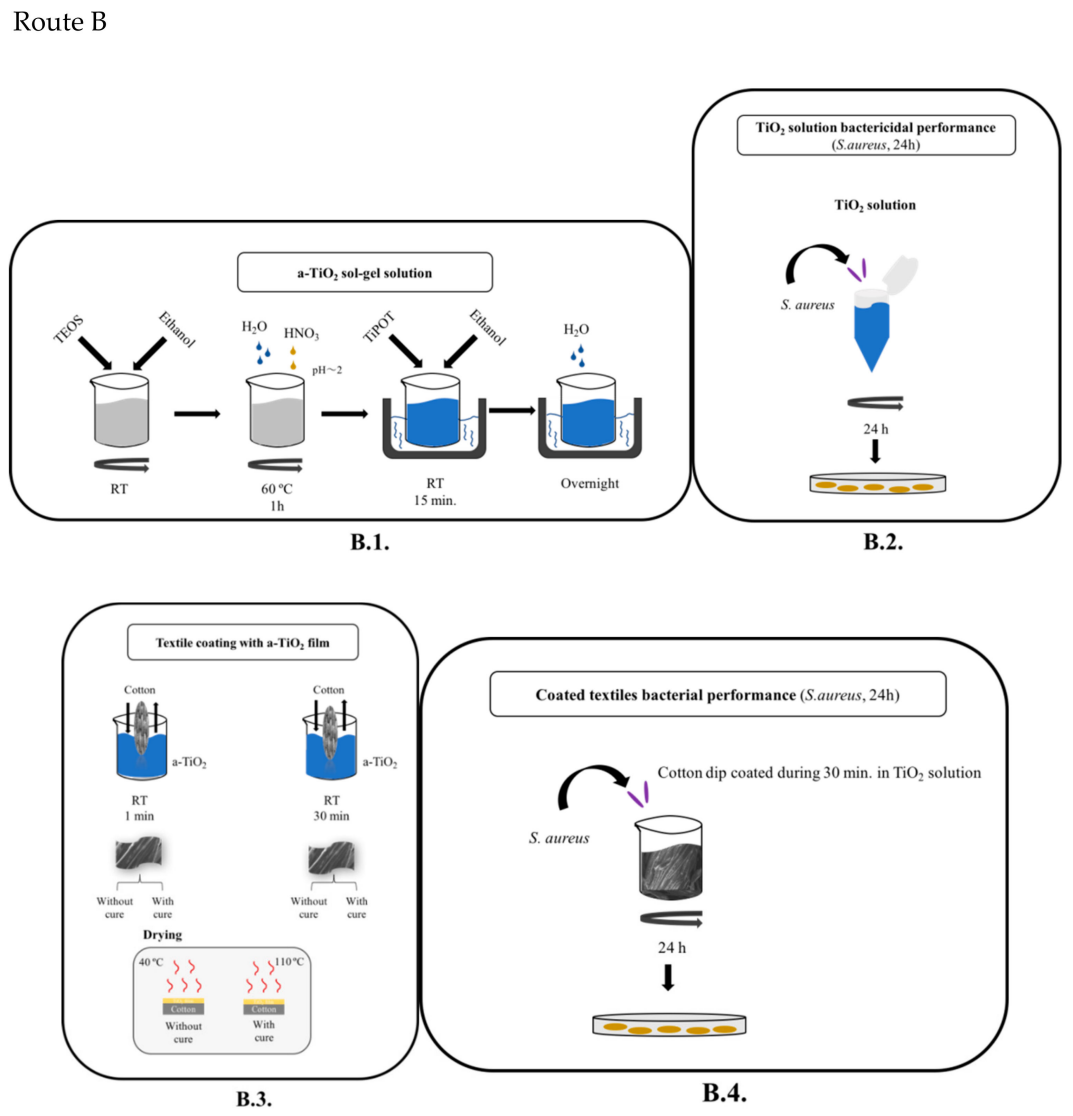
Process flowchart, namely: Route A (sol-gel NPs synthesis and textiles impregnation with NPs) and Route B (sol-gel film synthesis and textiles coating). Note: RT-room temperature; C-chitosan; TEOS-tetraethyl orthosilicate

The daylight bactericidal performance (without the need of external ultra-violet light source) of a-TiO_2_ (impregnated/coated) cotton (100% natural fibers) textiles was studied. Two main routes (A and B) were evaluated (Figure 2), namely:

Route A (Figure 2, Route A)
  a. Novel synthesis of a-TiO_2_ NPs (room temperature, sol-gel, base-catalyzed, eco-friendly protocol):
    a.1 pristine a-TiO_2_ NPs (A.1.)
    a.2 in situ amine-functionalized amorphous titania (a-TiO_2_-NH_2_) NPs (A.1.)
    a.3 ex situ CS-decorated amorphous titania (a-TiO_2_//CS) NPs (A.1.)
  b. Textile impregnation with a-TiO_2_ based NPs, to be industrially implemented as a finishing process (A.3.)
  c. Daylight bactericidal performance of NPs and impregnated cotton textiles according to ASTM E 2149 and AATCC 100 test methods using S. aureus (ATCC^®^6538TM) as bacterial indicator strain (A.2., A.4.)
Route B (Figure 2, Route B)
  d. TiO_2_ (acid-catalyzed) sol-gel solution preparation (B.1.)
  e. Textile coating with a-TiO_2_ film by dip in sol-gel solution, to be industrially implemented as a finishing process (B.3.)
  f. Daylight bactericidal performance of films and coated cotton textiles according to ASTM E 2149 and AATCC 100 test methods using S. aureus (ATCC^®^6538TM) as bacterial indicator strain (B.2., B.4.).

Steps b and e were performed with and without cure, so to test the role of temperature on the NPs’/films’ adhesion to the textile, according to the authors’ previous work [34]. The NPs were characterized by TEM, FTIR, and XRD, and were impregnated/coated textiles by SEM and FTIR.

#### 2.2.1. Amorphous Titania Nanoparticles Synthesis

In this work, pristine a-TiO_2_ NPs were synthesized for the first time through a room temperature, alkaline (base-catalyzed), and eco-friendly (ammonia free) sol-gel protocol, where hydrothermal water (SPA Cabeço de Vide, Cabeço de Vide, Portugal, pH ≈11) was used as a catalyst [34]. Briefly, a volume of 280 µL of sodium silicate solution (SSS; as nucleating agent) was diluted in 25 mL of absolute ethanol, and the resulting solution was placed under magnetic stirring for 15 min. A mixture of ethanol and hydrothermal water was then added to the suspension, and was stirred for 15 min. After this time, the suspension was placed in the ultrasound and 500 µL of titanium isopropoxide (TiPOT) was added, followed by 30 min of sonication.

In situ amine functionalization (a-TiO_2_-NH_2_ NPs) was performed according to the methodology described above. After titanium isopropoxide sonication (for 30 min), a volume of APTES was added and the mixture was left in magnetic stirring for 24 hours at room temperature. A molar ratio of 8:2 TiPOT to APTES was used. 

Chitosan decoration was performed ex situ (a-TiO_2_//CS NPs). The chitosan was solubilized in glacial acetic acid at 50 mM, based on the literature [24]. The NPs suspension was prepared by suspending 8 mg of a-TiO_2_ NPs (suspension 1) in 5 mL of absolute ethanol. Finally, 20 mL of CS (corresponding to 20 mg of CS) was added to the suspension. The mixture was left in magnetic stirring for 24 h at room temperature. The NPs and films acronyms and compositions are presented in Table 1.

#### 2.2.2. TiO_2_ Solution Preparation

A settled (acid-catalysed) sol–gel protocol was used for the preparation of the TiO_2_ sol–gel solution [33]. Briefly, 2.5 mL of TEOS was mixed with 1.5 mL of ethanol under magnetic stirring. Then, 0.25 mL of H_2_O (drop by drop) and two drops of HNO_3_ (or until pH ≈ 2) were added. The mixture was left in magnetic stirring for 1 h at 60 °C. After this time, 5 mL of ethanol and 0.83 mL of TiPOT were added. The mixture was placed under ultrasound for 15 min. Finally, 0.55 mL of bidistilled H_2_O was added to the mixture (drop by drop) and left overnight in magnetic stirring. 

The a-TiO_2_ textile film was prepared through dip-coating. The film acronyms and compositions are presented in Table 1.

#### 2.2.3. Textile Impregnation/Coating

The textile impregnation and coating were performed following the protocol (Figure 2):

1. Textile impregnation with amorphous titania-based NPs (a-TiO_2_, a-TiO_2_-NH_2_, and a-TiO_2_//CS; Figure 2A.3).
2. The textile impregnation was performed with 0.5% (in weight) on a cotton textile (according to the Portuguese Standard ISO 105-C06, 1994, see details in the literature [34]). Shortly, 25 mg of a-TiO_2_ NPs were suspended in 50 mL of distilled water, and were sonicated for 30 min. Then, 5 g of cotton textile (100% cotton) was immersed in the colloidal suspension, under magnetic stirring, at 40 °C for 15 min.
3. Textile coating with a titania solution (a-TiO_2_ film; Figure 2B.3).
4. Briefly, the cotton textile was immersed in the TiO_2_ sol–gel solution (at a constant rate of 2 cm/min) and then pulled up (also at the same rate). An a-TiO_2_ coating forms, its thickness being determined by the withdrawal rate (being inversely proportional). During the deposition and drainage steps, the solvent (alcohol) and the excess/unreacted reagents (water and/or silica/titania precursors) evaporate.
5. The impregnation/coating was performed without cure (woven at 40 °C, for 24 h) and with cure (at 110 °C, for 1h) to enhance the NPs’/films’ adhesion to the cotton textile [34].

### 2.3. Physical Characterization

#### 2.3.1. Transmission Electron Microscopy (TEM)

The morphology, size (static diameter), and size distribution of the synthesized a-TiO_2_-based NPs were assessed by transmission electron microscopy (TEM). A Hitachi H-8100 model was used, and the micrographs were obtained using an applied tension of 200 kV. This model is a conventional TEM with a high brightness LaB6 electron source and large specimen-tilt (>30°) capabilities. To prepare a TEM sample, a drop of the NPs suspension was placed on a copper grid and dried at room temperature.

#### 2.3.2. Fourier Transform Infrared Spectroscopy (FTIR)

Amorphous-TiO_2_-based NPs and powder films were analyzed by Fourier transform infrared spectroscopy (FTIR). FTIR was performed with potassium bromide pellets (KBr, ≥99+%, FTIR grade, from Sigma-Aldrich). Amorphous TiO_2_-based NPs and films were finely ground and mixed with potassium bromide, and then pressed into a disc (5 mg a-TiO_2_-based NPs or film to 200 mg KBr). The KBr pellet was used as a background. The Nicolet 5700 model was used in transmission mode, through a KBr beamsplitter.

#### 2.3.3. X-ray Powder Diffraction (XRD)

To confirm the amorphous character of the synthesized NPs and films, powder X-ray diffraction (XRD) was used. The diffractograms were obtained with a PANalytical X’Pert Pro diffractometer using Cu-Kα radiation. The data were collected in steps of 0.02° in the 20°–80° range (2θ), with a counting time per step of 4 s.

#### 2.3.4. Scanning Electron Microscopy (SEM)

Textile impregnation/coating efficiency was semi-quantitatively evaluated through field emission SEM. A JEOL JSM-7001F model, operated with a 15.0 kV accelerating voltage, was used. A simple random sampling (impregnated/coated cotton textile) without replacement was performed, and a minimum of 50 mm^2^ was observed for each set of textile nanocomposite (without and with cure). The textile samples were coated with a ≈80 nm layer of gold/palladium prior to the SEM observation.

### 2.4. Daylight Bactericidal Performance Evaluation

To determine the daylight bactericidal activity (without the need for an external ultra-violet light source) of the NPs/films and impregnated/coated textiles, assays based on the guidelines of the standard test methods of ASTM E 2149 and AATCC 100 were performed. The methods use a dynamic contact assay, where the immobilized antibacterial agent (a-TiO_2_-based NPs/films and impregnated/coated textiles) is incubated for 24 h with test strain cell suspensions (*S. aureus*, ATCC^®^6538^TM^ (Figure 2A.2,A.4,B.2,B.4). After incubation, the bacterial cells were plated and counted. Briefly, the procedure was carried out as follows: the bacterial cells of *S. aureus* were grown in Tryptic Soy Broth (TSB) for 24 h at 35 °C, and diluted using a KH_2_PO_4_ (0.3 mM) buffer solution to achieve a concentration from 1.5 × 10^5^ UCF/mL to 3 × 10^3^ UCF mL^−1^ (pH ≈ 7.3). The amount of NPs and film (in powder) tested in this assay was 0.061 ± 0.001 g, and 1 ± 0.1 g for the impregnated/coated textile samples (after weighting, the textile was finely cut). The samples were introduced in the Schott flaks, previously prepared, which were then placed in a wrist-shaker in daylight and at room temperature for 24 h (winter day cycle, 6 h day/18 h night). No artificial UV stimuli were induced. The bacterial reduction was calculated using a standard pour plate technique. At the beginning and end of the assay, 1 mL was drawn from each flask, doing successive dilutions of 1:10. The aliquots of each sample were seeded over culture plates with Luria agar (LA) medium in triplicates, which were then incubated for 20–24 h at 35 °C. For the determination of the bacterial reduction, the Colony Forming Units (CFU) were counted and the percentage of reduction, R (%), was quantified by the expression given in Equation (1). *C*_0_ represents the CFU of the control sample and C corresponds to the CFU of the analyzed material.

(1) $$R\;\left(\%\right)=\;\frac{\left(C_{0}-C\right)}{C_{0}}\;\times 100\%$$

The control assays were performed by incubating the bacterial cells in the absence of any kind of TiO_2_ or chitosan material. Briefly, the bacterial cells were incubated with the following: (i) buffer solution (negative control), (ii) NPs without a bactericidal performance or textile matrix with a reference bactericidal (positive control), and finally (iii) TiO_2_-based NPs/film under analysis. Triplicate assays were performed.

## 3. Results and Discussion

This work presents, for the first time, a room temperature, (eco-friendly) alkaline route sol–gel synthesis of TiO_2_-based NPs. Pristine a-TiO_2_ NPs, in situ amine-functionalized a-TiO_2_-NH_2_ NPs, and ex situ CS decorated a-TiO_2_//CS NPs (Table 1) were the synthesized compositions. Amine groups were used as the bonding strategy between a-TiO_2_ NPs and cotton textiles [34]. The chitosan (CS) polymer should provide an even higher surface bonding capacity towards single-handed a-TiO_2_-NH_2_ NPs, because of the presence of the amine, acetate, and hydroxyl groups. Nevertheless, CS was primarily chosen to reinforce the bactericidal performance of TiO_2_, according on the synergetic effects reported in the literature [20,21,22,23,24,25,26].

### 3.1. NPs Characterization

**Figure 3:**
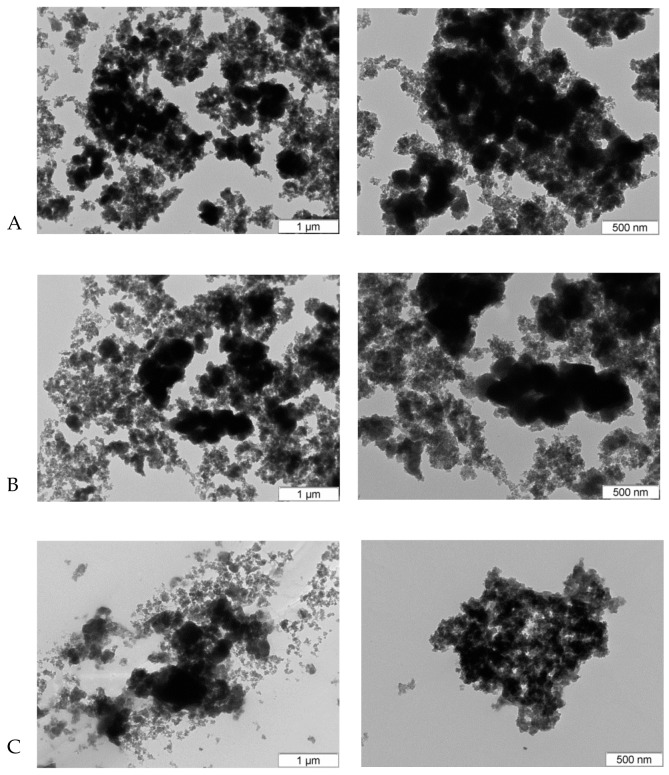
Transmission electron microscopy (TEM) images of a-TiO_2_-based nanoparticles (NPs), namely: (**A**) a-TiO_2_ NPs, (**B**) a-TiO_2_-NH_2_NPs, and (**C**) a-TiO_2_/CS NPs. (for each composition two different amplifications are shown—1 μm and 500 nm).

The surface area to volume ratio is central when it comes to surface properties. The NP size, size distribution, and morphology were studied. Figure 3A–C shows the TEM images of a-TiO_2_, a-TiO_2_-NH_2_, and a-TiO_2_//CS NPs, respectively. These images show that the synthesized NPs are monosized and spherical (static diameter ≈3–4 nm ± 0.1 nm), with the spherical morphology being the first sign of the amorphous character of TiO_2_ NPs [35].

**Figure 4:**
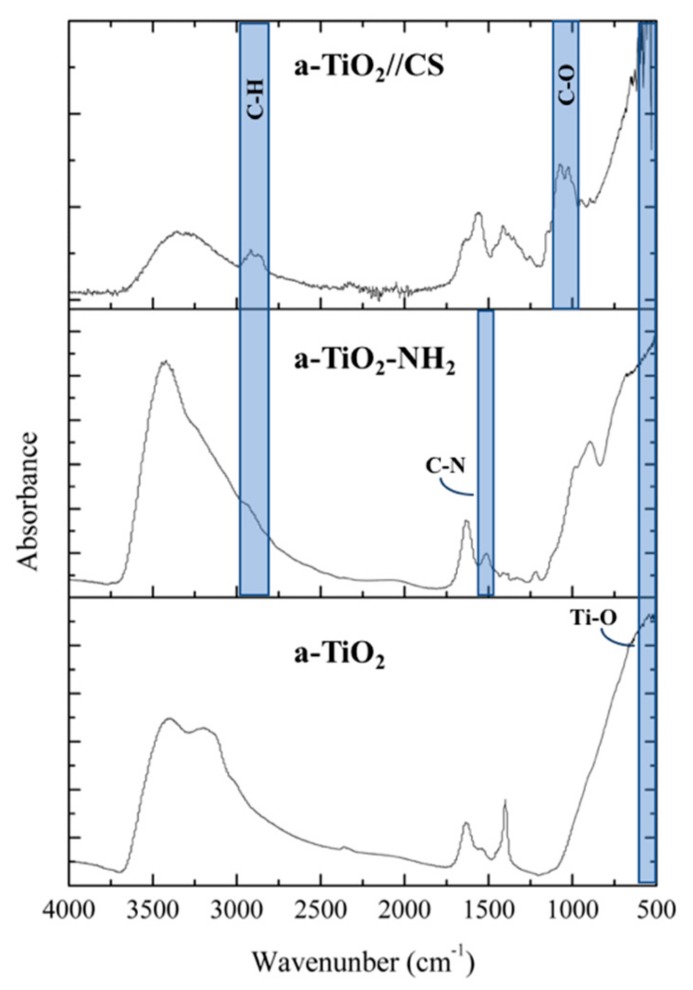
Fourier transform infrared spectroscopy (FTIR) spectra of a-TiO_2_-based NPs.

FTIR prove the presence of TiO_2_, amine groups and CS (Figure 4). The main FTIR TiO_2_ peak, centered at 526 cm^−1^ and assigned to the vibration of Ti-O bond [36], is present in all spectra, although no peaks assigned to Ti-O-Ti (600-400 cm^−1^, ≈550 cm^−1^, ≈342 cm^−1^) [36,37,38] were detected. In the case of a-TiO_2_-NH_2_ NPs spectrum a peak at 1510 cm^-1^, assigned to C-N bonds, and a slight shoulder at ≈2940 cm^−1^, assigned to C-H stretching were observed, proving the a-TiO_2_ NPs amine-functionalization. A-TiO_2_-NH_2_ NPs (the only hybrid matrix, TiPOT:APTES 8:2) does not exhibit typical Si-O-Ti peak at ≈949 cm^−1^ [36]. In the FTIR spectra of a-TiO_2_//CS NPs peaks in the range 1070–1020 cm^−1^ and 2900 cm^−1^ are assigned to C-O and C-H stretching, respectively, confirming the presence of CS polymer.

**Figure 5:**
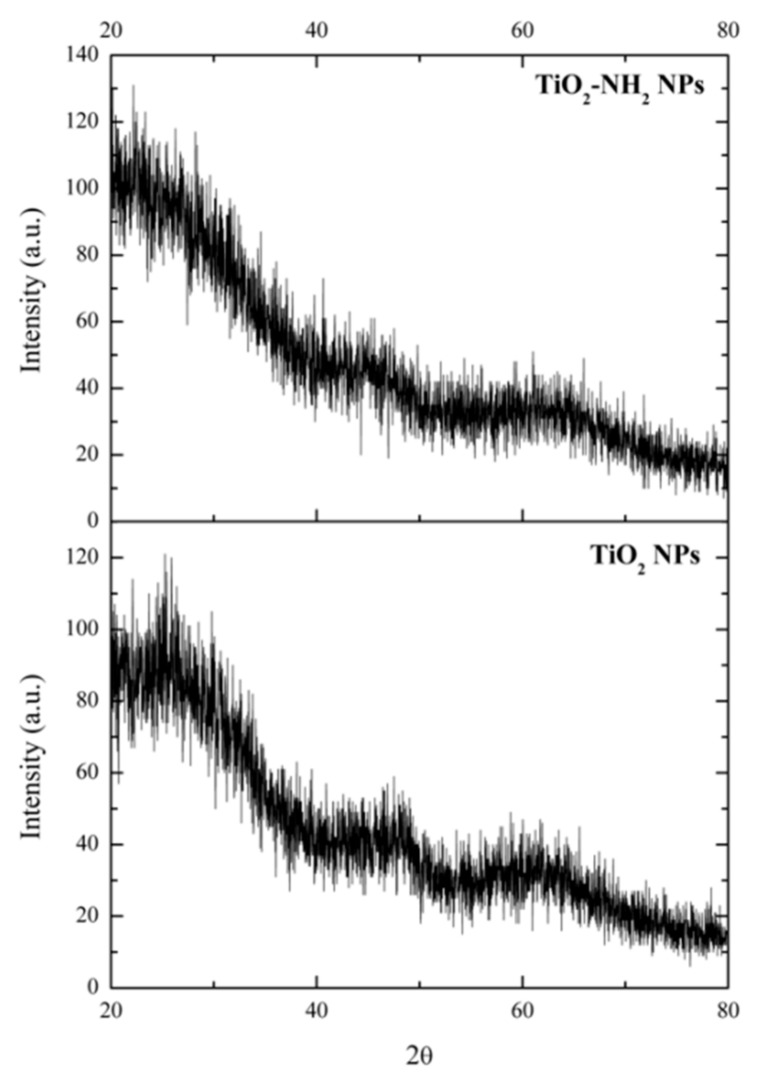
X-ray analysis (XRD) diffractograms of a-TiO_2_ and a-TiO_2_-NH_2_ NPs.

XRD characterizations confirm the amorphous character of all of the TiO_2_ NPs (a-TiO_2_, a-TiO_2_-NH_2_, and a-TiO_2_//CS) and films (Figure 5), as no defined or sharp peaks were observed in the diffractograms. Chitosan ex situ decoration of the a-TiO_2_ NPs did not affect the NPs’ amorphous character.

### 3.2. NPs Textile Impregnation Efficiency 

**Figure 6:**
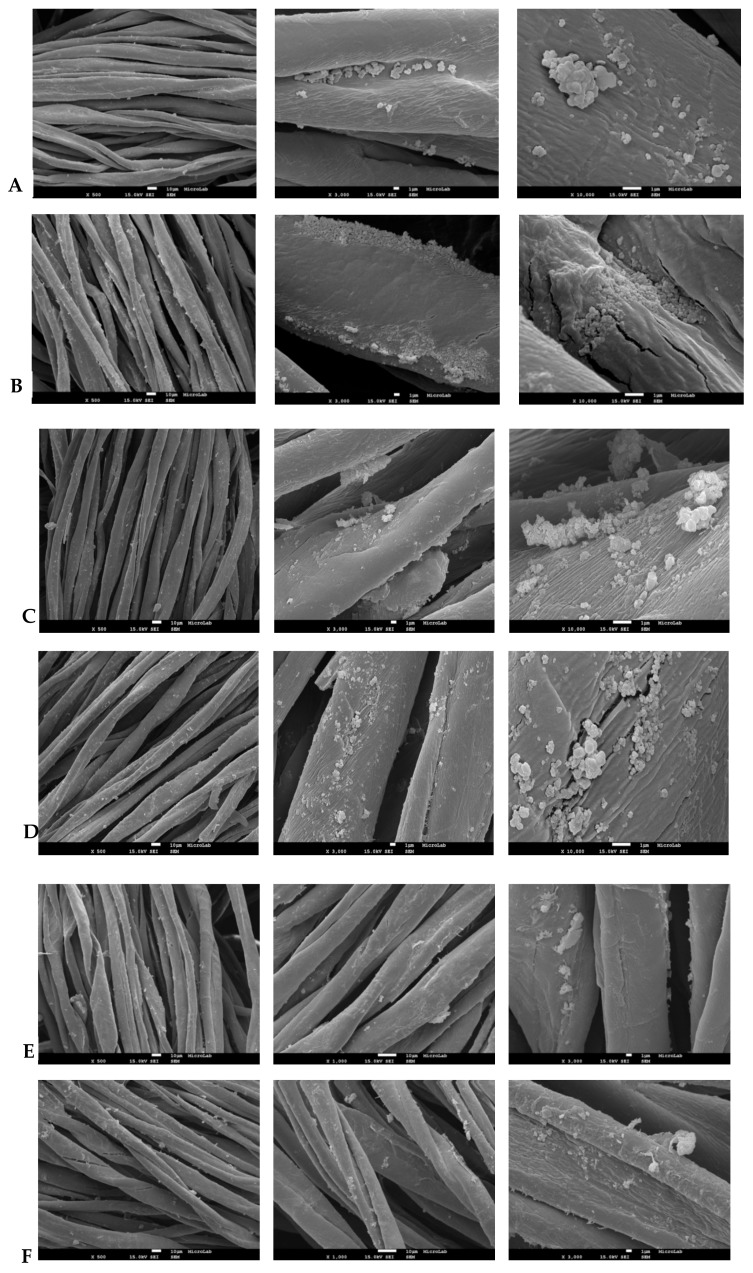
SEM images of cotton matrix impregnated with the following: a-TiO_2_ NPs (**A**-without cure; **B**-cured); a-TiO_2_-NH_2_ NPs (**C**-without cure; **D**-cured), and a-TiO_2_//CS NPs (**E**-without cure; **F**-cured).

All of the studied a-TiO_2_-based NPs bind to the cotton textile. Figure 6 shows the SEM images of the cotton textile impregnated with amorphous pristine (A and B) and amine-functionalized (C and D) titania NPs. 

Compared to pristine a-TiO_2_ NPs, a higher impregnation efficiency of amino-functionalized NPs (a-TiO_2_-NH_2_ NPs) was expected, in accordance with the author’s previous work [34]. The cure process is also supposed to enhance the NPs bidding efficiency [34]. Notwithstanding, in the present study, the amine-functionalization or cure did not boost the binding efficiency of the NPs to the textile.

Figure 6E,F also presents the SEM images of the cotton textiles impregnated with a-TiO_2_//CS NPs (cure and without cure). Compared to a-TiO_2_, a more efficient matrix coverage (higher number of NPs per unit area) was achieved. Here, the amine, acetate, and hydroxyl groups from the CS polymer do play an active role in cotton adhesion (through second order chemical bonding), offering higher bonding possibilities. Regarding the cure step, the SEM images reveal no significant difference between the cured and non-cured samples.

### 3.3. Film Textile Coating Efficiency

**Figure 7:**
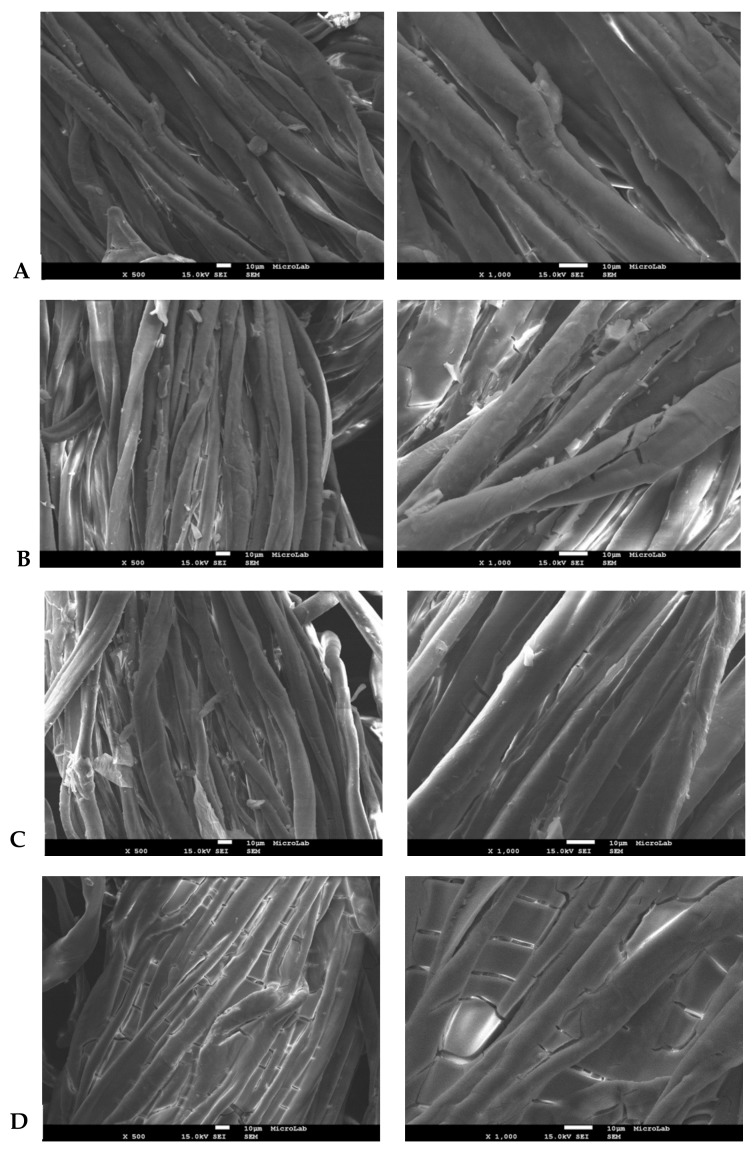
Cotton matrix coated with a-TiO_2_ film, as follows: after 1 min immersion (**A**-without cure; **B**-cured) and after 30 min immersion (**C**-without cure; **D**-cured).

Figure 7 shows a cotton textile coated with an a-TiO_2_ film, where a complete coverage of the textile matrix was successfully achieved. The immersion time did not play any role in cotton textile coverage (1 min and 30 min) efficiency.

### 3.4. Daylight Bactericidal Performance of NPs/Films and Impregnated/Coated Textiles

*S. aureus* is a major bacterial human pathogen that may cause a large variety of clinical symptoms. *S. aureus* transmission is typically from direct contact, from infections common in community-acquired and hospital-acquired settings [39,40].

**Figure 8:**
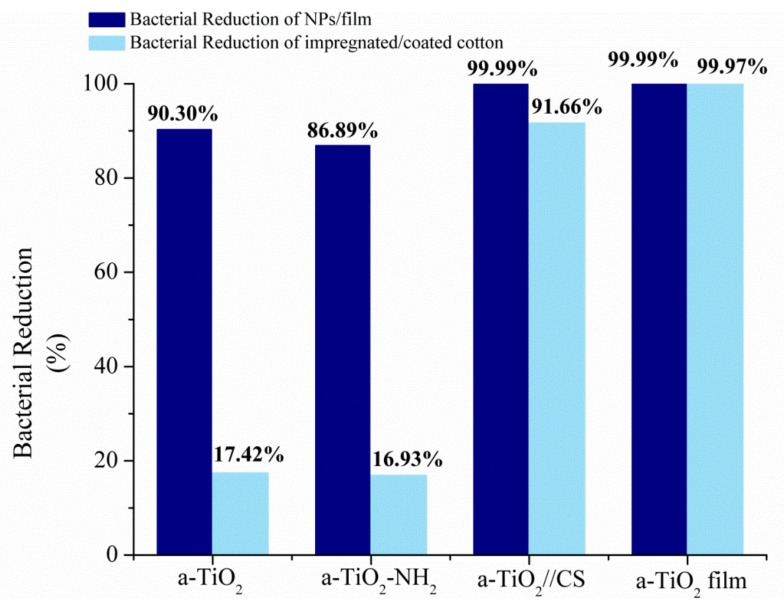
Daylight bacterial reduction of *S. aureus* achieved by a-TiO_2_ NPs/films and impregnated/coated cotton textiles.

In this work, the bactericidal properties of a-TiO_2_-based NPs were determined after 24h of incubation with the *S. aureus* cell suspension (ASTM E 2149 test method) at daylight (without any UV light exposition or stimuli), and by the determination of the cell reduction values for each of the NPs tested. Amorphous TiO_2_ NPs exhibited an antimicrobial efficiency above 90% of bactericidal reduction (Figure 8). Amine-functionalization slightly decreases the NPs bactericidal performance to 86.89%, which is attributed to NPs agglomeration (Figure 6C,D) and amine steric effects [41], both reducing the interface area between the NPs and bacteria.

Chitosan has been conjugated with a-TiO_2_ NPs to test the synergistic effect on the viability of *S. aureus*. CS decoration raised the bacterial performance to 99.99% (Figure 6), in accordance with the reported results in (crystalline) titania-chitosan complexes [28,42].

The a-TiO_2_-based NPs/films showed daylight bactericidal efficiency in the following order:

a-TiO_2_ film ≈ a-TiO_2_//CS NPs > a-TiO_2_ NPs > a-TiO_2_-NH_2_ NPs.

Regarding the textiles impregnated/coated with a-TiO_2_ NPs/films, the bactericidal performance was also determined after 20–24 h of incubation with the bacterial cell suspension of *S. aureus* (AATCC 100 test method). The tests were performed with natural daylight (24 h, natural day cycle of 8 h day/16 h night), without the use of any artificial UV lamps or any UV stimuli (during or before to the tests).

A bactericidal reduction of 17.42% was observed in the cotton textiles impregnated with a-TiO_2_ NPs, representing an enormous loss of bactericidal efficiency towards the equivalent NPs (90.30% bacterial reduction). When it comes to the textiles impregnated with amine-functionalized NPs, a bacterial reduction of 16.93% was observed, replicating the previous discouraging behavior. Only the assay performed with TiO_2_//CS impregnation exhibited a successful reduction on the bacterial cell colonies, of 91.66% (Figure 6). In the textiles impregnated with CS-TiO_2_ NPs, any coverage inefficiency will be outstripped by the CS-TiO_2_ synergetic bactericidal performance. 

The cotton textile coated with a a-TiO_2_ film exhibited the highest daylight bactericidal activity with a >99.97% bacterial reduction. Here, the complete textile coverage proves the high daylight bactericidal activity of the a-TiO_2_ structures. It may be concluded that the coverage ratio plays a determinant role in the textile’s bactericidal activity.

Titania cotton textiles showed a daylight bactericidal efficiency in the following order: 

a-TiO_2_ film > a-TiO_2_//CS NPs >>> a-TiO_2_ NPs ≈ a-TiO_2_-NH_2_ NPs.

## 4. Conclusions

Daylight bactericidal cotton textiles were developed, aimed at hospital use. Amorphous titania NPs were synthesized through a room temperature, base catalyzed, eco-friendly, easy-scalable sol–gel process, addressing textile impregnation during industrial finishing processes. Titania film synthesis followed the classical acid catalyzed route. Successful textile impregnation with a-TiO_2_-based NPs and textile coating with titania film drive to titania-cotton matrixes.

The daylight bactericidal performance of the NPs/films and impregnated/coated cotton textiles were validated according to the AATCC 100 and ASTM E2149 test methods, using *S. aureus* (ATCC^®^6538^TM^) as the bacterial indicator strain. The titania NPs’ and films’ daylight bactericidal efficiency was observed according to the following order: a-TiO_2_ film ≈ a-TiO_2_//CS NPs > a-TiO_2_ NPs > a-TiO_2_-NH_2_ NPs, following the sequence for the titania textiles, namely: a-TiO_2_ film > a-TiO_2_//CS NPs >>> a-TiO_2_ NPs ≈ a-TiO_2_-NH_2_ NPs.

The absence of any UV light or stimuli during the laboratorial bactericidal tests should be emphasized. Amorphous titania NPs/films and impregnated/coated textiles exhibited a bactericidal performance in natural daylight, being an industrially promising, constituting a cost-effective and technological affordable disinfection method that may be particularly useful in hospital and health services units. Furthermore, it is important to highlight that (i) amorphous oxides (NPs and films) usually present higher water and body fluids solubility, and therefore a lower toxicity, than the correspondent crystalline forms (which are commonly insoluble in water and body fluids, due to their lower Gibbs free energy values, discussed in the literature [43]), and (ii) the impregnation method simulates the domestic washing machine cycle, allowing for an easy, low-cost hospital re-charge of textiles.

## Figures and Tables

**Table 1:** Amorphous titania (a-TiO_2_)-based nanoparticles (NPs) and films acronyms, compositions, and sol-gel precursor’s volumes. CS-chitosan; APTES-3-aminopropyltriethoxysilane; TiPOT-titanium IV isopropoxide.

Acronym	Precursors	V_TiPOT_:V_Prec_(μL)	Catalyst
a-TiO_2_ NPs	TiPOT	500	Hydrothermal water pH ≈ 11
a-TiO_2_-NH_2_ NPs	TiPOT:APTES 8:2	TiPOT: 417 APTES: 83	Hydrothermal water pH ≈ 11
a-TiO_2_ film	TiPOT	830	Nitric acid pH ≈ 2
Acronym	Decoration	NPs:Decoration amount	
a-TiO_2_/CS NPs	Chitosan	10 mg NPs + 25 mg CS	
